# Experimental creation of quantum Zeno subspaces by repeated multi-spin projections in diamond

**DOI:** 10.1038/ncomms13111

**Published:** 2016-10-07

**Authors:** N. Kalb, J. Cramer, D. J. Twitchen, M. Markham, R. Hanson, T. H. Taminiau

**Affiliations:** 1 QuTech, Delft University of Technology, P.O. Box 5046, Delft 2600 GA, The Netherlands; 2 Kavli Institute of Nanoscience, Delft University of Technology, P.O. Box 5046, Delft 2600 GA, The Netherlands; 3 Element Six Innovation, Fermi Avenue, Harwell Oxford, Didcot, Oxfordshire OX11 0QR, UK

## Abstract

Repeated observations inhibit the coherent evolution of quantum states through the quantum Zeno effect. In multi-qubit systems this effect provides opportunities to control complex quantum states. Here, we experimentally demonstrate that repeatedly projecting joint observables of multiple spins creates quantum Zeno subspaces and simultaneously suppresses the dephasing caused by a quasi-static environment. We encode up to two logical qubits in these subspaces and show that the enhancement of the dephasing time with increasing number of projections follows a scaling law that is independent of the number of spins involved. These results provide experimental insight into the interplay between frequent multi-spin measurements and slowly varying noise and pave the way for tailoring the dynamics of multi-qubit systems through repeated projections.

## Abstract (web summary)

Repeated observations of quantum states inhibit coherent evolution through the Zeno effect, providing opportunities for controlling multi-qubit systems. Here the authors demonstrate that projecting joint observables of three spins in diamond creates quantum Zeno subspaces that suppress dephasing.

## Graphical abstract

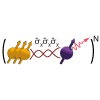

The quantum Zeno effect restricts the evolution of repeatedly observed quantum systems. For a two-dimensional system the state simply is frozen in one of two eigenstates of the measurement operator^1^,^2^,^3^,^4^,^5^,^6^,^7^,^8^,^9^,^10^. In multi-dimensional systems; however, Zeno subspaces are formed that can contain complex quantum states and dynamics: repeated observations create a barrier that blocks coherent evolution between subspaces, but leaves coherences and dynamics within those subspaces intact^11^. Analogous effects can also be realized through coherent control pulses or strong driving fields that decouple transitions between the subspaces^12^,^13^,^14^,^15^,^16^,^17^,^18^,^19^. Pioneering experiments have highlighted that the non-trivial dynamics in Zeno subspaces can be used to prepare exotic quantum states^20^,^21^,^22^,^23^,^24^. However, the opportunities to tailor the dynamics of multi-qubit systems by restricting coherent evolution have remained unexplored.

Here we show that repeated multi-spin projections on individually controlled spins create quantum Zeno subspaces that can encode multiple logical qubits while suppressing dephasing caused by the environment. We realize these repeated projections for up to three nuclear spins in diamond using the optical transition of a nearby electron spin. We then encode up to two logical qubits—including entangled states of logical qubits—and show that increasing the frequency of the projections supresses the dephasing of quantum states. Finally, we theoretically derive and experimentally verify a scaling law that shows that the increase in dephasing time is independent of the number of spins involved.

## Results

### Experimental system and sequence

Our system consists of three ^13^C spins (*I*=½) surrounding a single nitrogen vacancy (NV) centre (|0〉_*NV*_ : *m*_*s*_=0 and |1〉_*NV*_ : *m*_*s*_=−1) in diamond (see Supplementary Note 1). The natural evolution of the ^13^C spins is dominated by dephasing due to the slowly fluctuating surrounding bath of ^13^C spins (dephasing times 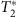
=12.4(9), 8.2(7) and 21(1) ms for spin 1, 2 and 3, respectively)^25^. Because the fluctuations are quasi-static, the Hamiltonian in a given experiment is 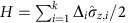, with *k* the number of spins and the detuning Δ_*i*_ for spin *i* drawn from a Gaussian distribution with 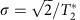. We denote the Pauli operators as 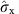, 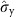, 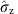 and the identity as 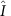.

The quantum Zeno effect arises when an observable 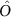 is projected (super-operator 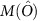). Here we consider dichotomic observables with eigenvalues ±1. A projection leaves the system's density matrix (*ρ*_s_) in block-diagonal form with respect to the projectors 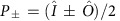 (ref. 11):


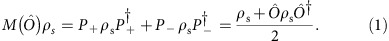


**Figure 1:**
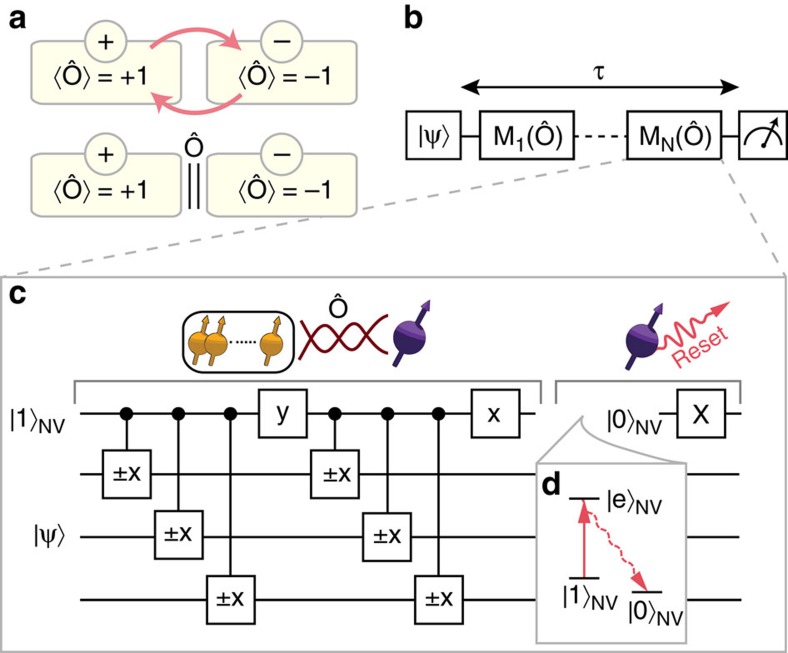
Concept and experimental sequence. (**a**) Quantum Zeno subspaces. The state space of a quantum system is divided into two subspaces (yellow boxes) of an observable 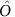. Plus and minus signs indicate eigenvalues of the associated operator. Coherent transitions between the two subspaces occur while the system is unperturbed (top, red arrows) but are strongly inhibited if 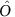 is repeatedly projected (bottom). (**b**) Experimental sequence. After initialization in |*ψ*〉, *N* equidistantly distributed projections *M*(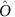) (see equation (1)) are applied during a total evolution time *τ* and the state of the system is read out. (**c**) Realization of 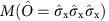 for three nuclear spins. First, the state of the nuclear spins (yellow) is entangled with the ancilla electron-spin state (purple). Second, the electron spin is projected and reinitialized in |1〉_NV_ (see also **d**) through a long 30 μs optical pumping pulse to |0〉_NV_ and a subsequent microwave *π*-pulse (X). Such a long laser excitation pulse ensures that the NV is projected. The *x* and *y* gates are *π*/2 rotations around the *X* and *Y* axes, respectively. Controlled gates indicate that the direction is determined by the electron spin^28^. See Supplementary Fig. 3 for pulse sequences for projections on one and two spins. (**d**) Relevant electron spin levels for optical re-pumping through selective resonant excitation of |1〉_NV_ to |*e*〉_NV_. We prepare the nuclear spin states in the 〈
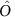
〉=+1 subspace and associate this subspace with the electron state |0〉_NV_ in the entangling sequence so that the optical projection ideally never excites the NV centre.

Repeatedly projecting observable 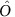 thus inhibits coherent evolution between the two eigenspaces of 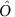. We choose joint multi-spin observables of the form 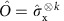, which anti-commute with all terms in the Hamiltonian *H*, so that rapid projections ideally result in the effective Zeno Hamiltonian 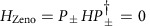 (ref. 11). Applying these projections therefore suppresses dephasing for each nuclear spin, but leaves quantum states and driven dynamics inside the two subspaces untouched (Fig. 1a).

To investigate quantum Zeno subspaces we use the following experimental sequence (Fig. 1b). We first initialize the nuclear spins in the desired state and prepare the electron spin in |1〉_NV_. Crucially, leaving the electron in |1〉_NV_ creates a different frequency shift for each of the three ^13^C spins that suppresses resonant flip-flop interactions among the ^13^C spins during idle time^26^. We then apply a total of *N* projections that are equally distributed in time. Finally, the nuclear spin state is read out using the electron spin as an ancilla (refs 27, 28, 29, 30, 31, 32). Here we consider the case of an even number of projections. The results for an odd number of projections *N* give rise to additional effects at long evolution times due to the time-correlations in the noise and are discussed in Supplementary Fig. 1. The total evolution time *τ* is defined from the end of the initialization to the start of the read-out. We subtract the time that control operations are applied to the nuclear spins (averaged over all spins), as dephasing might be suppressed during driving (for a comparison see Supplementary Fig. 2).

We experimentally realize repeated multi-spin projections on the ^13^C spins by using the NV electron spin as an ancilla spin (Fig. 1c). First, we entangle the NV electron spin state with the projections on the eigenspaces of 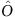 (〈
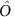
〉=+1 or −1), so that the combined state is 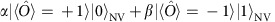 (refs 25, 33). Second, we apply an optical excitation that is resonant only if the electron-spin state is |1〉_NV_ (‘reset')^25^, which projects the quantum state and re-initializes the NV electron spin in |0〉_NV_ through optical pumping (Fig. 1d). Note that it is not required to extract or record the outcome of the optical measurement. To mitigate extra dephasing caused by the stochastic nature of the optical re-initialization (time constant of ∼1 μs), we use ^13^C spins with a NV-^13^C hyperfine coupling that is small compared with the inverse of the time constant for re-initialization (all couplings are below 2*π*·50 kHz)^34^,^35^. In addition, we design the gate sequence, so that |0〉_NV_ is associated with the subspace of the initial nuclear state: ideally the electron spin is never optically excited and the projection constitutes a null measurement.

### Quantum Zeno effect for a single spin

**Figure 2:**
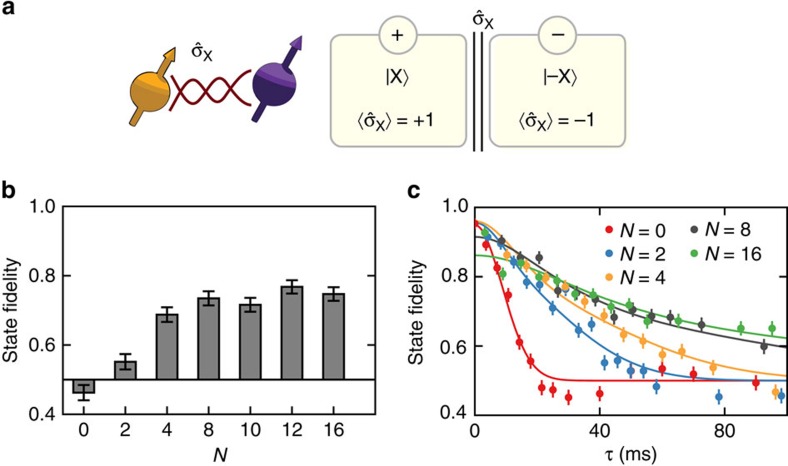
Quantum Zeno effect for a single-spin superposition state. (**a**) Quantum Zeno subspaces for a single nuclear spin (spin 1) and 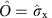. Each eigenspace of 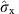 consists of one state (|*X*〉 or |−*X*〉) with the respective eigenvalue indicated by the circled +/− signs. (**b**) State fidelity for |*X*〉 after *τ*=40 ms. The fidelity initially increases with the number of projections *N*. (**c**) The complete time traces for the storage of |*X*〉 show that the dephasing time increases with the number of projections. The curves are fits to the theoretically expected fidelity (see equation (3)). All data are corrected for the final read-out fidelity (Supplementary Fig. 4 and Supplementary Note 3). All error bars are 1 s.d.

To illustrate the quantum Zeno effect and to benchmark our system, we first consider a single ^13^C spin and study the dephasing of the superposition state 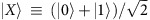 for 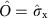 (Fig. 2a). We initialize the ^13^C spin in |*X*〉 with an initial state fidelity of 0.95(2) and apply up to *N*=16 projections. For a fixed total evolution time of 40 ms, we observe a significant increase of the state fidelity with an increasing number of projections (Fig. 2b). The complete time traces show that the dephasing time increases as more projections are applied (Fig. 2c); the superposition state is protected by the quantum Zeno effect. In this example, however, the Zeno subspaces contain just a single state and therefore cannot encode general quantum states.

### Preserving a logical qubit via quantum Zeno subspaces

**Figure 3:**
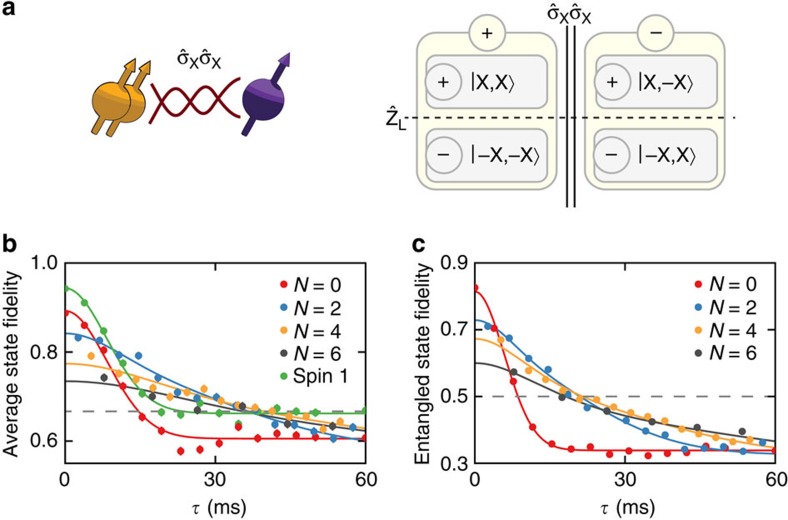
Storing a logical quantum bit by repeated two-spin projections. (**a**) Schematic representation: the four-dimensional state-space of two ^13^C spins (spin 1 and 2) is divided into two subspaces by repetitively projecting 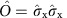 through entanglement with the ancilla spin. We define a logical quantum bit with logical operator 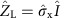 (dashed line) and associate 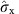 in 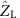 with the spin with the longest coherence time (spin 1). (**b**) Storing a logical quantum bit. The average logical state fidelity for the six logical input states, for example, 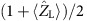 for |0〉_L_, as a function of time and for a varying number of projections *N*. To compare the results to the best possible decay for a single nuclear spin, we compare to the individual decay of spin 1 and eliminate potential systematic detunings by measuring 
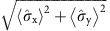
 (instead of 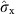 or 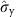). The dashed horizontal line is the classical limit of 2/3 (ref. 37). (**c**) Preserving two-spin entangled states. The two-spin state fidelity, averaged over the four entangled input states, indicates that general two-spin states in the subspace are preserved. Above the dashed horizontal line (*F*=0.5) the state is entangled. For *N*=2, 4 and 6 projections, entanglement is preserved longer than without projections. Solid lines are fits to equation (3) with the initial amplitude *A*, an offset and the effective dephasing time 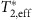 as free parameters. Error bars are 1 s.d. and are smaller than the symbols.

We next investigate Zeno subspaces that can contain an arbitrary two-dimensional quantum state, that is, a complete logical quantum bit, by performing joint projections on two ^13^C spins. We set the joint-observable 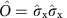, so that the four-dimensional state space is divided into two coherent two-level subspaces (Fig. 3a). In these subspaces a logical qubit, which can hold an arbitrary quantum state, can be defined as 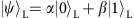, with 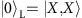 and 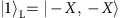, and with logical operators 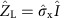 and 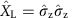. Note that logical qubit superposition states are generally entangled states of the two ^13^C spins.

We characterize the storage of arbitrary quantum states by preparing all six logical basis states 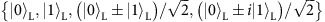 and averaging the final logical state fidelities^36^ (Fig. 3b). The logical qubit without projections shows the same decay as a single ^13^C spin, but with a slightly reduced initial fidelity (*F*=0.89(1)) due to the overhead of creating the entangled states |*ψ*〉_L_. Applying projections of the joint-observable 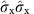 strongly suppresses the dephasing by the environment, while preserving the logical qubit states. As a result, the average state fidelity for the logical qubit surpasses the best ^13^C nuclear spin used, while still remaining above the threshold of 2/3 for the storage of quantum states^37^. This result demonstrates the suppression of the dephasing of a complete logical qubit through the quantum Zeno effect.

Interestingly, preserving the logical qubit does not actually require the coherence of the second spin to be maintained, as follows from the logical operator 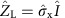. To show that the complete two-spin state is preserved, including entanglement between the two nuclear spins, we measure the average state fidelity with the ideal two-spin state for the four entangled initial states as a function of time (Fig. 3c). The duration for which genuine entanglement persists (two-spin state fidelity >0.5) is extended for *N*=2, 4 and 6 projections compared with the case without any projections, indicating that the barrier introduced by the projections inhibits dephasing for any two-spin state within the Zeno subspace.

### Quantum Zeno subspaces with two logical qubits

**Figure 4:**
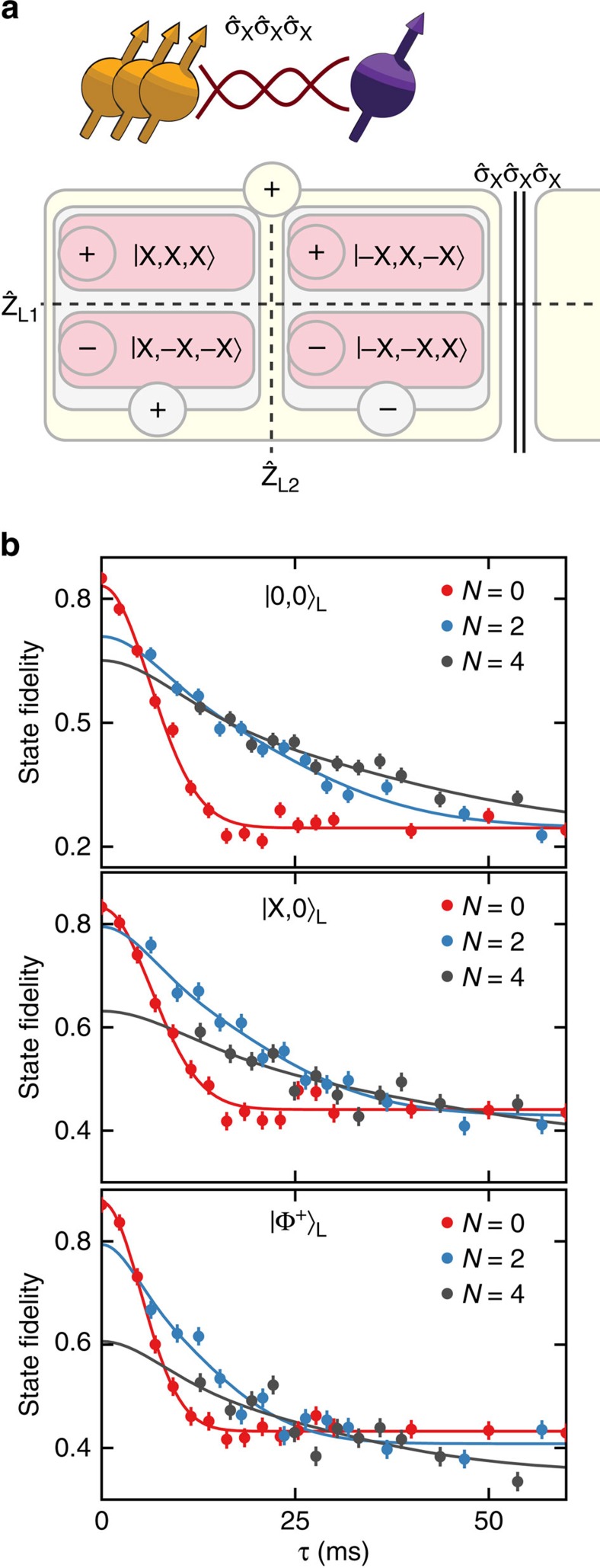
Two logical qubits in a quantum Zeno subspace. (**a**) Schematic representation for three nuclear spins (spins 1–3) and 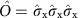. Two four-dimensional subspaces are created (yellow box). For simplicity we only show the positive subspace, which contains states of the form 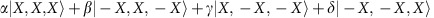. Within this subspace two logical qubits are defined by the logical operators 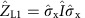, 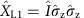 and 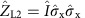, 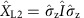 (blue and red boxes). (**b**) Logical state fidelities for three logical states: eigenstate |0,0〉_L_, superposition state |X,0〉_L_, and the entangled state |Φ^+^〉_L_. The results show that repeated projections of the three-spin operator 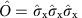 preserve the two logical qubits while inhibiting dephasing. Solid lines are fits to equation (3). The fidelities decay to different values for large τ because |Φ^+^〉_L_ and |X,0〉_L_ are eigenstates of operators of the form 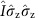 or one of its permutations, whose expectation values are unaffected by dephasing. Error bars are 1 s.d.

Realizing Zeno subspaces with even more dimensions enables the exploration of complex states of multiple logical qubits within the subspaces. We include a third nuclear spin and set 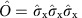 to create a protected four-dimensional subspace, which can host two logical qubits defined by the logical operators 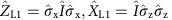 and 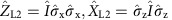 (Fig. 4a). Each pure state within the 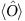
=+1 subspace can be expressed in terms of the logical two-qubit states:


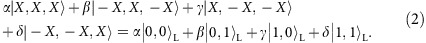


To investigate the inhibition of dephasing of the two logical qubits by repeated projections we prepare three different logical states: the logical eigenstate state |0,0〉_L_, the logical superposition state 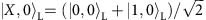 and the entangled logical state 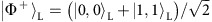. Preserving this set of states requires repeated projections of the three-spin operator 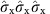, since they are not eigenstates of a single two-spin operator.

The logical state fidelities for all three states show a clear prolongation of the decay times for *N*=2 and 4 three-spin projections (Fig. 4b). Moreover, for a range of evolution times, the absolute logical state fidelities are increased despite the initial loss of fidelity due to the complexity of the experimental sequence (33 two-qubit gates for *N*=4, which in total require 1,276 refocusing pulses on the electron spin). These results confirm that the introduced three-spin projections inhibit dephasing of the individual spins while preserving the two logical qubits in a quantum Zeno subspace.

### Scaling law for the suppression of dephasing

To gain a detailed quantitative understanding of the quantum Zeno effect for multi-spin projections, we derive a complete analytical description for the evolution. We model the projections as instantaneous and the noise as a quasi-static Gaussian frequency detuning, independent for each nuclear spin. We find an analytic solution for the decay of the expectation value of observables that are sensitive to dephasing (for *N* projections and total evolution time *τ*):


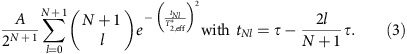


Here *A* ≤ 1 is the initial amplitude determined by experimental fidelities and 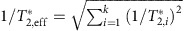 is an effective joint decay rate of all involved spins. This result is valid for any system size, that is, number of spins, and number of projections *N* (both even and odd). A detailed derivation of equation (3) is given in Supplementary Note 2.

**Figure 5:**
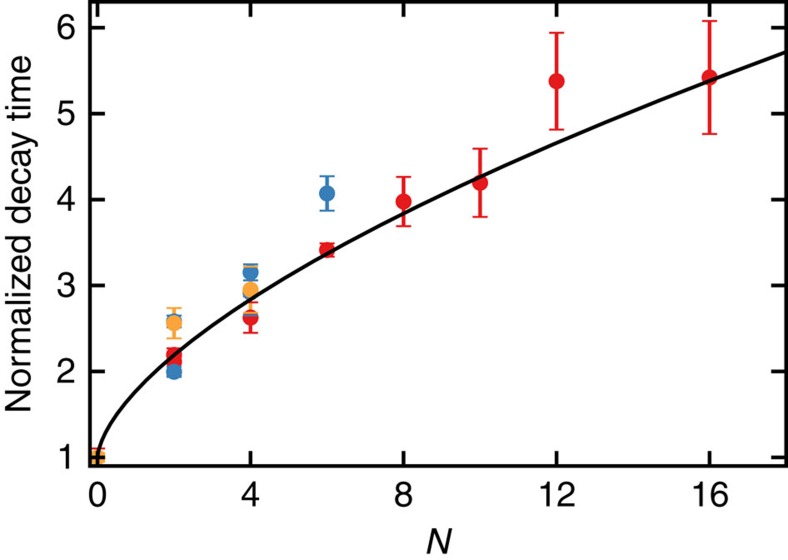
Scaling of the decay time with increasing number of projections. The fitted decay times for all measurements in this article (Figs 2, 3, 4) are compared with the theoretical decay time enhancement (solid line). All values are taken relative to the value without projections (*N*=0). The data are averaged according to the number of operators in the expectation values that are subject to dephasing (that is, the number of 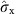 and/or 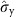). Red: one operator. Blue: two operators. Orange: three operators (see Supplementary Fig. 5 for raw data). To show that the normalized decay time is independent of the number of nuclear spins, we distinguish data with a differing total number of nuclear spins. For instance, measurements of 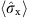 (with 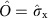) or 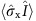 (with 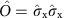) are represented by separate data points. The theory curve is obtained by evaluating equation (3) up to *N* = 16 (see Supplementary Fig. 6). The obtained curve depends only on the functional shape of the underlying quasi-static noise spectrum. Error bars are 1 s.d.

We fit all experimental data in Figs 2, 3, 4 with *A*, 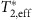 and an offset, to account for the fact that two out of six cardinal states are insensitive to dephasing, as free parameters. We find good agreement with the experimentally obtained dephasing curves (see Supplementary Table 1 for all fit values). To analyse the increase of the decay time with increasing number of projections, we compile the extracted values from all experiments with an even number of projections and with 1–3 nuclear spins in Fig. 5. The results reveal a scaling law that is independent of the number of spins involved, in good quantitative agreement with our theoretical model.

## Discussion

In conclusion, we have observed that repeatedly projecting joint-observables of multi-spin systems creates quantum Zeno subspaces that can hold complex quantum states, and that these Zeno subspaces are resilient to environmental dephasing. While suppression of dephasing may also be achieved through alternative techniques such as coherent refocusing^17^,^18^,^19^, our results provide direct experimental insight into the physics of repeated multi-spin measurements and Zeno subspaces in low-frequency noise environments. The results are also of practical relevance in the context of quantum error correction and detection codes, in which errors are detected through repeated measurements of joint observables^25^,^38^,^39^. Moreover, the demonstrated methods pave the way for investigating the effect of repeated measurements in various noise environments, for example, non-Markovian noise, and for exploring and engineering complex dynamics of multi-qubit systems under tailored decoherence^40^,^41^,^42^,^43^.

### Data availability

The data that support the findings of this study are available from the corresponding author upon request.

## Additional information

**How to cite this article:** Kalb, N. *et al*. Experimental creation of quantum Zeno subspaces by repeated multi-spin projections in diamond. *Nat. Commun.* **7,** 13111 doi: 10.1038/ncomms13111 (2016).

## Supplementary Material

Supplementary Information Supplementary Figures 1-6, Supplementary Table 1, Supplementary Notes 1-3 and Supplementary References.
