# Supplementary material for: Experimental creation of quantum Zeno subspaces by repeated multi-spin projections in diamond
Source: Nat Commun. 2016 Oct 7;7:13111. doi: 10.1038/ncomms13111 (PMC5059787; doi:10.1038/ncomms13111)
Supplement: Supplementary Information — Supplementary Figures 1-6, Supplementary Table 1, Supplementary Notes 1-3 and Supplementary References. [file ncomms13111-s1.pdf]

SUPPLEMENTARY FIGURES

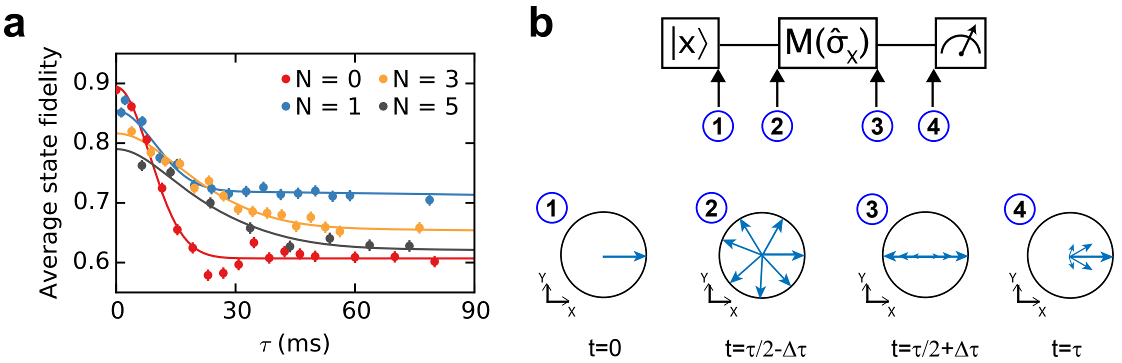

Supplementary Figure 1 Analysis for an odd number of projections. (a) Experimental results for the logical qubit in the two-spin case (Fig. 3b of the main text). We observe a clear elevation of the average state fidelity for times much longer than T_2^* for 1 (blue) and 3 (orange) projections. This was also observed in J. Cramer et al. for $N = 1$ [1]. The behavior for times much longer than the decoherence time is fully explained by our model and can be accurately fit with Supplementary Eq. (5). (b) Schematic explanation of the elevated signal for large evolution times $\tau \gg T_2^*$. Top panel: A spin-1/2 particle is initialized in a superposition state $|X\rangle$, evolves freely for a time $\tau/2$ until it is projected $(M(\hat{\sigma}_x))$ onto $|\pm X\rangle$ and is finally read out after a total evolution time of τ. Bottom panel: Sketch of an ensemble of states in the XY plane of the Bloch sphere. The state of the particle is well defined after initialization ①. The state is however completely mixed due to a random frequency detuning before projection ② if the free evolution $\tau/2$ is much longer than the dephasing time T_2^*. Projection into $|\pm X\rangle$ effectively projects the Bloch vector onto the x-axis ③. The frequency detuning of the particle remains constant after projection such that the ensemble average partly rephases (similarly to a spin echo) after another evolution time of $\tau/2$. We call this effect a filter since a single projection of $\hat{\sigma}_x$ completely mixes the state if the particle is in $|\pm Y\rangle = (|X\rangle \pm i\,|-X\rangle)/\sqrt{2}$, effectively filtering those cases out. In contrast states which are affected by a detuning but got rotated to $|\pm X\rangle$ at the time of the projection are unaffected. All error bars are 1 s.d.

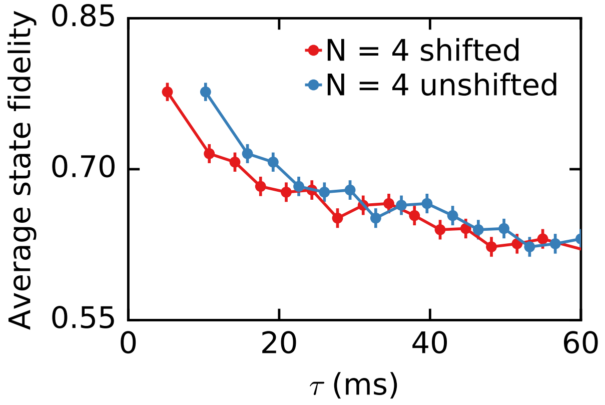

Supplementary Figure 2 As stated in the main text, the average driving time is subtracted from the evolution time since dephasing might be largely suppressed during driving. Here we provide a comparison of data with subtracted average driving time (red) and an unshifted data set (blue). The applied shift is 5.06 ms. The shown data corresponds to the data in Fig. 3b of the main text with $N = 4$. All error bars are 1 s.d.

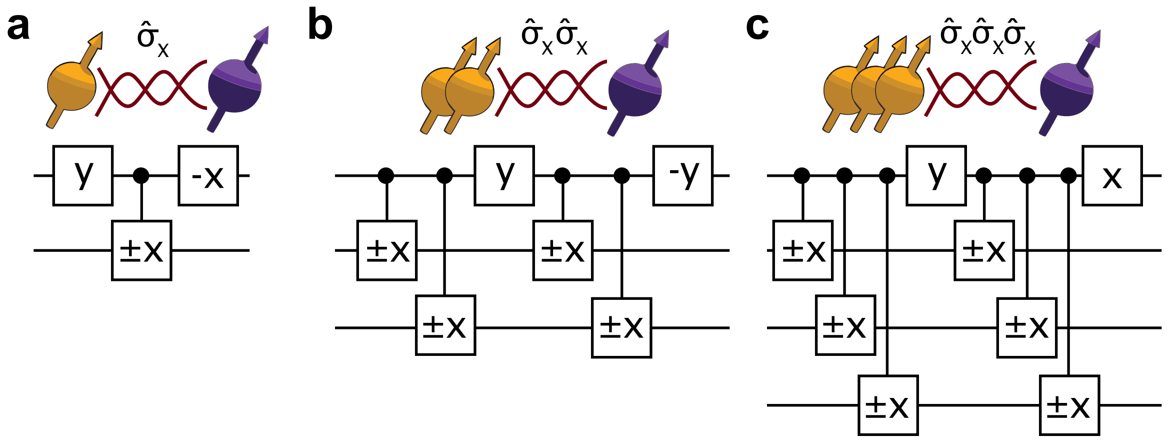

Supplementary Figure 3 Gate sequences to project the observables $\hat{\sigma}_x$ (a), $\hat{\sigma}_x\hat{\sigma}_x$ (b) and $\hat{\sigma}_x\hat{\sigma}_x\hat{\sigma}_x$ (c). x and y are $\pi/2$ rotations around the X and Y axis with the orientation given by the sign. We use a combination of dynamical decoupling sequences that act as rotations on the ^{13}C nuclear spins with the sign controlled by the electron spin [3] (controlled gates in all panels). Compared to a standard CNOT these gates cause an extra unconditional $\pi/2$ rotation. We pre-compensate this extra rotation with the gates before the first gate on the electron spin. Note that for a single spin (panel a) all eigenstates ($|\pm X\rangle$) of the projected observable are unaffected by the extra operation and one can therefore omit the compensation.

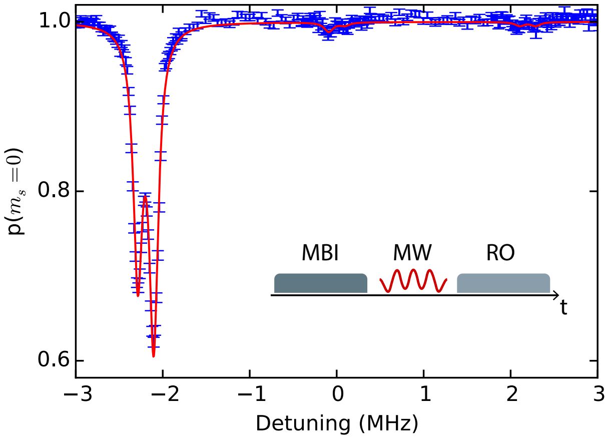

Supplementary Figure 4 Electron spin resonance to determine the initialization fidelity of the ^{14}N nuclear spin ($I = 1$) in $m_I = -1$. Inset: Schematic of the experimental sequence. After measurement-based initialization of the nucleus (MBI) a weak microwave pulse (MW) [2] with a duration of $8\,\mu$s is applied and followed by optical read out (RO) of the electron spin. The detuning is relative to the central microwave frequency (1.74667(1) GHz) of the $m_s = 0$ to $m_s = -1$ transition of the electron spin. The transition is split into six lines due to strong hyperfine coupling to the ^{14}N nuclear spin (with a coupling strength of $2\pi \cdot 2.195(1)$ MHz) and a ^{13}C nuclear spin ($I = 1/2$, with a coupling strength of $2\pi \cdot 182(1)$ kHz) in the vicinity of the NV centre. The data is therefore fit to six Lorentzian functions with variable width, spacing and amplitude. From the fitted amplitudes we extract the population of the ^{14}N nuclear spin after MBI: $p_{-1} = 0.96(1)$ $p_0 = 0.022(8)$ $p_{+1} = 0.014(8)$. All error bars are 1 s.d.

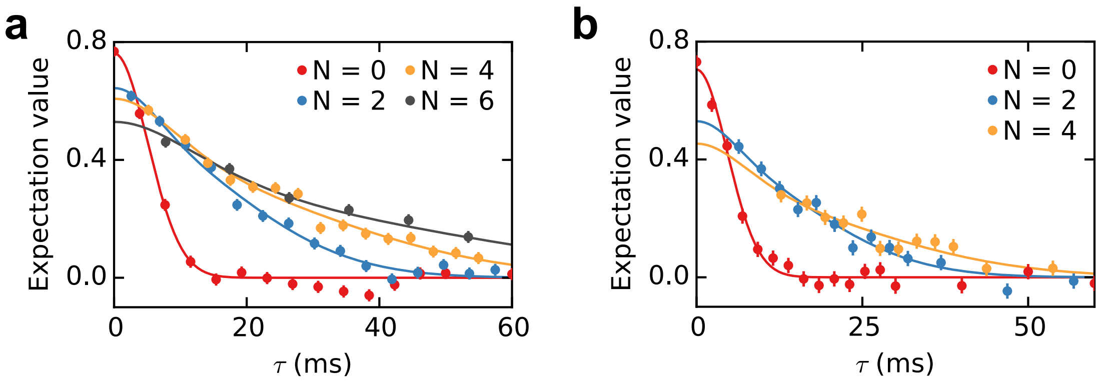

Supplementary Figure 5 (a) Decay of $\langle\hat{\sigma}_x\hat{\sigma}_x\rangle$ for a varying number of projections. The data are averaged over six input states that correspond to the states used for Fig. 3b of the main text. The number of projections N is given in the legend. (b) Decay of $\langle\hat{\sigma}_x\hat{\sigma}_x\hat{\sigma}_x\rangle$. The data are averaged over the three states of Fig. 4b of the main text. The fitted $1/\sqrt{e}$-time for $N = 0$ is 4.6(2) ms. The amount of projections for each data trace is given in the legend. The fitted, relative, decay constants for these data are included in Fig. 5 of the main text. All error bars are 1 s.d.

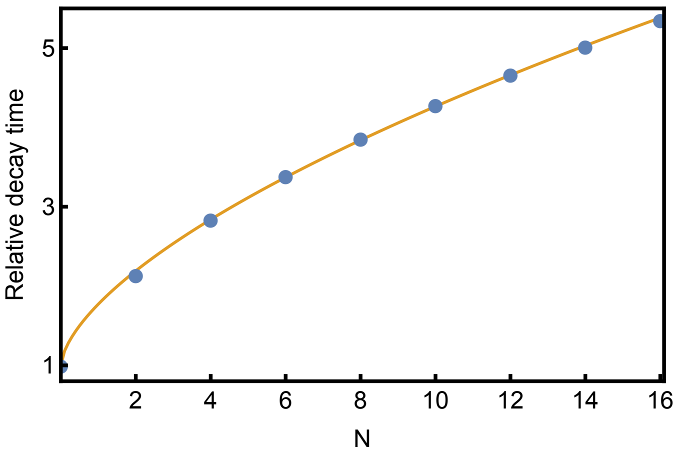

Supplementary Figure 6 Blue data: extracted $\frac{1}{\sqrt{e}}$-time from the analytical solutions to Supplementary Eq. (5). Orange line: Fitting the extracted times to the power law $1 + \mu M^{\nu}$ yields $\mu = 0.77(1)$ and $\nu = 0.63(1)$ and gives a good approximation for the experimentally investigated number of projections N.

	fitted T_2^* (ms)	expected $T_{2,eff}^*$ (ms)
C_1	12.4(9)	/
C_2	8.2(7)	/
C_3	21(1)	/
C_1C_2	7.1(3)	6.8(3)
$C_1C_2C_3$	6.5(2)	6.5(4)

Supplementary Table I Comparison of fitted and expected decay time T_2^* for multi-qubit observables. The expected T_2^* value is calculated by assuming uncorrelated noise and using $\left(1/T_{2,\mathrm{eff}}^*\right)^2 = \sum_i \left(1/T_{2,i}^*\right)^2$ (see Supplementary Note 3). We find agreement between expected and measured decay times.

SUPPLEMENTARY NOTE 1: DEVICE CHARACTERISTICS

The experiments were conducted in a confocal microscope at cryogenic temperatures (4 K). The investigated sample is a chemical-vapour-deposition homoepitaxially grown diamond of type IIa with a natural composition of carbon isotopes. The diamond has been cut along the $< 111 >$ crystal axis and was grown by Element Six.

SUPPLEMENTARY NOTE 2: ANALYTICAL MODEL AND DATA PROCESSING

1. Derivation of the analytical model

This section outlines the derivation of Eq. (3) of the main text. We consider k two-level systems with random, uncorrelated and constant detuning Δ_i for each two-level system. Without loss of generality, the initial ($t = 0$) state is chosen to be the balanced superposition state $|X_1 \cdots X_k\rangle$. This state is an eigenstate of the projected operator $\hat{\sigma}_{\mathrm{x},1} \cdots \hat{\sigma}_{\mathrm{x,k}}$. After the first projection at time t and evolution of the system for another time t an analytic expression for the expectation value $\langle \hat{\sigma}_{\mathrm{x},1} \cdots \hat{\sigma}_{\mathrm{x,k}} \rangle_{N=1} = \mathrm{Tr}\left[\rho \hat{\sigma}_{\mathrm{x},1} \cdots \hat{\sigma}_{\mathrm{x,k}}\right]$, with the density matrix ρ, can be derived.

$$\langle \hat{\sigma}_{\mathrm{x},1} \cdots \hat{\sigma}_{\mathrm{x,k}} \rangle_{N=1} = \frac{1}{2^k} \sum_{\alpha_1 \in \{-1,+1\}} \cdots \sum_{\alpha_N \in \{-1,+1\}} \cos^2[(\sum_{i=1}^{k} \alpha_i \Delta_i)t] = \frac{1}{2^{k-1}} \sum_{\alpha_2 \ldots \alpha_k} \cos^2[(\Delta_1 + \sum_{i=2}^{k} \alpha_i \Delta_i)t] \,. \tag{1}$$

With the sum over all possible combinations of relative detunings by choosing the binary values of $\alpha_i = \pm 1 \;\forall\; i \in \{1, 2, ..., k\}$. Note that the notation for the sum over all configurations of α_i has been simplified for the last equality. This sum of cosine terms originates from static terms in the relevant entries of the density matrix after projection. The formula for a single projection ($N = 1$) is then readily extended to N projections

$$\langle \hat{\sigma}_{\mathrm{x},1} \cdots \hat{\sigma}_{\mathrm{x,k}} \rangle_N = \frac{1}{2^{k-1}} \sum_{\alpha_2 \ldots \alpha_k} \cos^{N+1}[(\Delta_1 + \sum_{i=2}^{k} \alpha_i \Delta_i)t]. \tag{2}$$

We obtain the ensemble average $\overline{\langle \hat{\sigma}_{x,1} \cdots \hat{\sigma}_{x,k} \rangle}_N$ by integration over a normal distribution $G_i[\Delta_i]$ of width $\sigma_i = \sqrt{2}/T_{2,i}^*$ for each Δ_i

$$\overline{\langle \hat{\sigma}_{\mathrm{x},1} \cdots \hat{\sigma}_{\mathrm{x,k}} \rangle}_N = \int \cdots \int \langle \hat{\sigma}_{\mathrm{x},1} \cdots \hat{\sigma}_{\mathrm{x,k}} \rangle_N \prod_{i=1}^{k} G_i[\Delta_i] d\Delta_i. \tag{3}$$

We single out one summand of $\langle \hat{\sigma}_{\mathrm{x},1} \cdots \hat{\sigma}_{\mathrm{x,k}} \rangle_N$ with general α_i and perform the integration. The $\cos^{N+1}$ term is rewritten by using Euler's formula and the Binomial theorem

$$\cos^{N+1}[(\Delta_1 + \sum_{i=2}^{k} \alpha_i \Delta_i)t] = \frac{1}{2^{N+1}} \sum_{l=0}^{N+1} \binom{N+1}{l} \exp\left[i(\Delta_1 + \sum \alpha_i \Delta_i))t\right]^{N+1-2l} . \tag{4}$$

After inserting this rewritten cosine term into Supplementary Eq. (3) we obtain a product of Fourier transformations for each summand in Supplementary Eq. (4). It becomes clear that the precise assignment of the α_i coefficients does not play a role for the evaluation of the integral since the Fourier transformation and inverse Fourier transformation of a Gaussian give the same result: i.e. the precise assignment of $\alpha_i = \pm 1$ does not play a role for the evaluated integral. The normalization factor of $\frac{1}{2^{k-1}}$ therefore drops out when summing over all possible configurations of α_i. Evaluating Supplementary Eq. (3) results in an analytic expression for the ensemble average $\overline{\langle \hat{\sigma}_{\mathrm{x},1} \cdots \hat{\sigma}_{\mathrm{x,k}} \rangle}_N$

$$\overline{\langle \hat{\sigma}_{\mathrm{x},1} \cdots \hat{\sigma}_{\mathrm{x,k}} \rangle}_N = \frac{1}{2^{N+1}} \sum_{l=0}^{N+1} \binom{N+1}{l} \exp\left[-((N+1-2l)t)^2 \sum_{i=1}^{k} (1/T_{2,i}^*)^2\right] . \tag{5}$$

Supplementary Eq. (5) describes the expected decay curve for a joint k-partite observable after N joint projections. All operations are separated by the same duration t. Involving multiple nuclear spins results in an effective decay time $(1/T_{2,\mathrm{eff}}^*)^2 = \sum_i (1/T_{2,i}^*)^2$ (as expected from the convolution of two Normal distributions). Correlations which are only partially subject to dephasing, e.g. $\langle \hat{\sigma}_{\mathrm{z},1} \hat{\sigma}_{\mathrm{y},2} \rangle$, only incorporate the relevant decoherence times (for the given example: $T_{2,\mathrm{eff}}^* = T_{2,2}^*$). In the main text, t is replaced by the total evolution time $\tau = (N+1)t$.

2. Fitting routine

The fits to the data are performed in the following way. First, the decay without projections ($N = 0$) is fit with a Gaussian function. The initial amplitude, offset and width are extracted. Second, in order to fit data sets with multiple projections Supplementary Eq. (5) is multiplied with the extracted amplitude and the offset is added (the offset originates from constant $\langle \hat{\sigma}_{\mathrm{z},1} \cdots \hat{\sigma}_{\mathrm{z,k}} \rangle$ correlations that are not subject to dephasing and play a role when determining average state fidelities). The data set is then fitted with three free parameters: $T_{2,\mathrm{eff}}^*$, a global amplitude damping that parametrizes errors due to the added complexity of the experiment and the aforementioned constant offset.

3. Scaling law

In order to obtain the theory curve in Fig. 5 of the main text, we compute the theoretical enhancement of the dephasing time for a given number of projections. We calculate the normalized $\frac{1}{\sqrt{e}}$-time of Supplementary Eq. (5) for N being even and smaller than 17. A modified scaling law, $1 + \mu N^{\nu}$, is fit to the extracted characteristic dephasing times and the parameters $\mu = 0.77(1)$ and $\nu = 0.63(1)$ (see Supplementary Fig. 6) are found.

SUPPLEMENTARY NOTE 3: ^{13}C SPIN READ-OUT CORRECTION

We correct the read-out results for errors introduced by the final conditional gates on the ^{13}C spins to obtain the actual state fidelity. We employ a characterization technique developed in reference [1] and determine correction factors (C_{C_i}) for one-, two- and three-spin expectation values. The applied correction assumes a symmetric initialization and read-out process as well as a constant loss of fidelity due to imperfect initialization of the ^{14}N spin of the NV centre. The probability to find the ^{14}N spin in $m_I = -1$ after initialization is found to be 0.96(1) (see Supplementary Fig. 4).

We obtain the following correction factors

$$
\begin{aligned}
C_{C_1} &= 0.94(1) & C_{C_2} &= 0.94(1) & C_{C_1C_2} &= 0.93(2) \\
C_{C_1C_2C_3} &= 0.90(2) & C_{C_1C_3} &= 0.93(2) & C_{C_2C_3} &= 0.95(2).
\end{aligned} \tag{6}
$$

SUPPLEMENTARY REFERENCES

[1] Cramer, J., N. Kalb, M. A. Rol, B. Hensen, M. S. Blok, M. Markham, D. J. Twitchen, R. Hanson, and T. H. Taminiau (2016), Nat. Commun. **7**, 11526.

[2] Pfaff, W., B. J. Hensen, H. Bernien, S. B. van Dam, M. S. Blok, T. H. Taminiau, M. J. Tiggelman, R. N. Schouten, M. Markham, D. J. Twitchen, and R. Hanson (2014), Science **345**, 532.

[3] Taminiau, T. H., J. Cramer, T. van der Sar, V. V. Dobrovitski, and R. Hanson (2014), Nat. Nano. **9**, 171.
